# Adenosine triphosphate drives head and neck cancer pain through P2X2/3 heterotrimers

**DOI:** 10.1186/2051-5960-2-62

**Published:** 2014-06-05

**Authors:** Yi Ye, Kentaro Ono, Daniel G Bernabé, Chi T Viet, Victoria Pickering, John C Dolan, Markus Hardt, Anthony P Ford, Brian L Schmidt

**Affiliations:** Bluestone Center for Clinical Research, New York University, 421 First Avenue, 233 W, New York, NY 10010 USA; Department of Oral Maxillofacial Surgery, New York University, New York, NY 10010 USA; Technology and Automation Operations, Bioanalytical Sciences, Genentech, South San Francisco, CA 94080 USA; Department of Orthodontics, New York University, New York, NY 10010 USA; Department of Applied Oral Sciences, The Forsyth Institute, Cambridge, MA 02142 USA; Afferent Pharmaceuticals, San Mateo, CA 94403 USA

**Keywords:** ATP, P2X2, P2X3, Mechanical allodynia, Cancer pain

## Abstract

**Introduction:**

Cancer pain creates a poor quality of life and decreases survival. The basic neurobiology of cancer pain is poorly understood. Adenosine triphosphate (ATP) and the ATP ionotropic receptor subunits, P2X2 and P2X3, mediate cancer pain in animal models; however, it is unknown whether this mechanism operates in human, and if so, what the relative contribution of P2X2- and P2X3-containing trimeric channels to cancer pain is. Here, we studied head and neck squamous cell carcinoma (HNSCC), which causes the highest level of function-induced pain relative to other types of cancer.

**Results:**

We show that the human HNSCC tissues contain significantly increased levels of ATP compared to the matched normal tissues. The high levels of ATP are secreted by the cancer and positively correlate with self-reported function-induced pain in patients. The human HNSCC microenvironment is densely innervated by nerve fibers expressing both P2X2 and P2X3 subunits. In animal models of HNSCC we showed that ATP in the cancer microenvironment likely heightens pain perception through the P2X2/3 trimeric receptors. Nerve growth factor (NGF), another cancer-derived pain mediator found in both human and mouse HNSCC, induces P2X2 and P2X3 hypersensitivity and increases subunit expression in murine trigeminal ganglion (TG) neurons.

**Conclusions:**

These data identify a key peripheral mechanism in cancer pain and highlight the clinical potential of specifically targeting nociceptors expressing both P2X2 and P2X3 subunits (*e.g*., P2X2/3 heterotrimers) to alleviate cancer pain.

**Electronic supplementary material:**

The online version of this article (doi:10.1186/2051-5960-2-62) contains supplementary material, which is available to authorized users.

## Introduction

Despite recent advances in cancer treatment, pain control persists as an important clinical challenge, especially since cancer patients are living longer [1]. Among all cancers, head and neck (H&N) cancer is one of the most painful [2, 3]. H&N cancer pain typically locates at the primary site and significantly impairs speech, swallowing, and masticatory function [2, 4]. The peripheral mechanism underlying H&N cancer pain is not clearly understood.

In the peripheral nervous system, extracellular adenosine triphosphate (ATP) contributes to nociception by activation or sensitization of P2X receptors [5, 6]. P2X2 and P2X3 are mostly expressed in nociceptive sensory neurons and participate in transduction of painful signals [7]. P2X2 and P2X3 subunits can form homotrimeric P2X2, P2X3, or heterotrimeric P2X2/3 receptors, each with unique pharmacological properties. The different receptor subtypes might mediate different pathological pain conditions. Activation of P2X3 homotrimers by ATP leads to a transient current with rapid desensitization, whereas activation of P2X2 homotrimers by ATP leads to a sustained current; activation of P2X2/3 heterotrimers by ATP leads to a current with intermediate desensitization kinetics [8, 9]. In rodents P2X3 subunits are highly expressed on small-to-medium size sensory neurons, while P2X2 subunits are expressed on medium-to-large sensory neurons, and colocalization of P2X2 and P2X3 subunits (putative P2X2/3 heterotrimers) are mainly found on medium sized neurons [7]. It has been suggested that P2X3 homotrimers are responsible for acute pain, while P2X2/3 heterotrimers mediate chronic pain [10, 11].

Recent animal studies highlight a role for ATP and its purinergic receptors containing P2X3 subunits in bone cancer pain [12–15]. The selective P2X3, P2X2/3 receptor antagonist A-317491 transiently attenuates cancer-induced bone pain in mice, but has no effect at the late stage of bone cancer [13]. Bone cancer pain in rats is reduced by the blockade of P2X3 and P2X2/3 receptors with AF-353 [14]. However, these findings in animal models do not necessarily translate into human physiology [16] and there is no direct evidence to show whether ATP correlates with pain in cancer patients. In addition, oral cancer is distinct from bone cancer pain. Oral cancer produces pain at the primary site whether it involves the submucosa, muscle or jaws. Bone cancer pain is almost always due to metastasis from another primary site. The mechanism of ATP and purinergic signaling in oral cancer pain is not as well characterized as it is in the bone cancer pain model. Whereas P2X2 and P2X3 subunit immunoreactivities are readily detectable in dorsal root ganglia (DRGs) and TG and in tongues of rats and mice [17–21], human DRG reportedly express P2X3 but not P2X2 mRNA [16], and human TG have not been studied for P2X2 expression. Furthermore, the cancer microenvironment contains many other mediators such as NGF [22], that could affect P2X2 and P2X3 expression, pharmacology and electrophysiological response in sensory neurons [18, 23, 24]. Increased expression of P2X3 subunits were reported in peripheral tissues as well as DRGs in bone cancer pain models [15, 25]. Compared to well-studied P2X3 subunits, the role of P2X2 subunits in cancer pain is less clear.

Here we used a translational approach investigating the contribution of peripheral ATP and the differential roles of P2X2 and P2X3 subunits in pain induced by HNSCC, a cancer notoriously painful in patients [4]. We further investigated whether NGF, a key tumor-derived mediator of HNSCC pain and proliferation [22], modulated P2X2 and P2X3 receptor expression, pharmacological function and electrophysiological response. Our findings have immediate implications on our understanding of cancer pain in patients, and the development of potentially beneficial pharmacological treatments targeted at the ATP/P2X system.

## Methods

The study was approved by the Institutional Review Board of New York University College of Dentistry and University of California San Francisco (UCSF). All patients provided written informed consent in accordance with the Declaration of Helsinki. Patients were enrolled with the following inclusion criteria: 1) biopsy-proven HNSCC, and 2) no history of prior surgical, chemotherapeutic, or radiation treatment for oral SCC. The validated UCSF Oral Cancer Pain Questionnaire [26] was administered to enrolled patients. The questionnaire consisted of eight questions on spontaneous and functional pain, which were rated on a visual analog scale (0 to 100 mm). None of the patients were taking analgesics or were receiving cancer treatment at the time of questionnaire completion. Demographic information was collected for each patient including age, sex, ethnicity, HNSCC location (tongue, floor of mouth, buccal mucosa, gingiva, palate), tumor size (greatest dimension based on clinical examination), and evidence of metastasis.

### Animals

Female athymic, immunocompromised (BALB/c nu/nu) mice and BALB/c mice were purchased from Charles River Laboratories. All experiments were performed according to the policies of the International Association for the Study of Pain and approved by the New York University Institution Animal Care and Use Committee.

### HPLC analysis

HNSCC and anatomically matched, contralateral normal oral epithelium from 13 oral cancer patients were surgically removed, immediately snap frozen in liquid nitrogen and stored at −80°C. ATP was quantified with HPLC coupled to UV detection. Each tissue sample was weighed and ground in 10% Trichloroacetic acid buffer (MP Biomedical). Samples were incubated on ice for 1 hour, and were periodically vortexed for 3 seconds. After incubation, samples were centrifuged for 15 min at 10000 rpm in 4°C. The supernatant was collected into pre-cooled centrifuge tubes and neutralized with 0.2 M K_2_HPO_4_. The sample was kept on ice for an additional 15 minutes to precipitate the insoluble salts and centrifuged again for 10 minutes at 10000 rpm at 4°C. The resulting supernatant was decanted into a collecting tube, drawn into a 1 ml syringe and passed through a 0.22 μM filter (Fisher Scientific). The samples were then transferred to vials and injected at a volume of 10 μl into the HPLC system equipped with a Waters 2795 Separations Module Microsampler (Waters Corporation). The mobile phase was pumped at 1 ml/min and consisted of 0.1 M KH_2_PO_4_, pH 5.0. Samples were passed through a 100 × 4.1 mm C18 column (Waters Corporation). A Waters 2487 Absorbance Detector was set at 260 nm (Waters Corporation). Identification and quantification of ATP in samples were accomplished using retention time and area under the curve produced by injecting an ATP standard (Sigma-Aldrich) into the HPLC system under identical conditions. ATP standard calibration concentration ranged from 0 to 500 μM. A good linear relationship was observed between ATP concentrations against area under the curve (R^2^ = 0.999) or peak height (R^2^ = 0.998). ATP concentration was calculated based on area under the curve and was normalized against the weight of each extracted tissue.

### Cell culture

#### Cancer cells

The human head and neck cancer cell line, HSC-3 (ATCC) derived from a human tongue SCC, was cultivated in Dulbecco’s Modification of Eagle’s Medium (DMEM) with 4.5 g/L glucose, l-glutamine and sodium pyruvate, supplemented with 10% fetal bovine serum (FBS), 25 μg/mL fungizone, 100 μg/mL streptomycin sulfate, and 100 U/mL penicillin G and cultivated at 37°C in 5% CO_2_.

#### Neurons

Mouse TG neurons were harvested and cultured as previously described [27]. Briefly, BALB/c mice were euthanized with isoflurane. Trigeminal ganglia were removed, transferred into HBSS and enzyme-digested by incubation with papain (Worthington), collagenase type II (CLS2) (Worthington), and dispase type II (MB). Dissociated neurons were plated on glass coverslips coated with poly-d-lysine and laminin and maintained for approximately 2 hr at 37°C at 5% CO_2_/95% air in F12 media (Gibco BRL) supplemented with 10% FBS.

#### Co-culture

Coverslips containing TG neurons were transferred into culture dishes containing HSC-3 cells in fresh F12 medium supplemented with 10% FBS and cultured for 1 day before co-culture experiments. Anti-NGF antibody (R&D Systems) was used at 50 ng/ml and added directly into culture medium.

### Mouse models

Six to eight week-old female athymic, immunocompromised (BALB/c nu/nu) mice and BALB/c mice were purchased (Charles River Laboratories). Mice were housed in a temperature-controlled room on a 12:12 light:dark cycle (0600–1800 h light), with *ad libitum* access to food and water. All experiments were performed according to the policies and recommendations of the International Association for the Study of Pain and approved by the New York University Institution Animal Care and Use Committee.

#### SCC supernatant model

HSC-3 cells were grown in 10 cm cell culture dishes to 90% confluency; the medium was changed to serum-free DMEM (2 mL volume), and incubated for 48 hours. Culture supernatant was then collected on the day of injection. 50 μl of SCC supernatant was injected into the right hind paw of BALB/c mice anesthetized with isoflurane. Control mice received the same volume of serum-free DMEM in the right hind paw. 3 mg/kg or 10 mg/kg A-317491 was directly dissolved into 50 μl SCC supernatant. AF-353 was first dissolved in DMSO (Sigma-Aldrich) and the solution was then added into 50 μl of SCC supernatant. Both A-317491 and AF-353 are strong antagonists to P2X3 and P2X2/3 receptors, and are weak antagonists to P2X2 receptors. AF-353 is a more potent antagonist, with high oral bioavailability and CNS penetration [14, 28].

#### Paw SCC model

A paw cancer pain model was created by inoculating 10^6^ HSC-3 cells, suspended in vehicle consisting of 50 μl volume DMEM and Matrigel™ (Becton Dickinson & Co), into the right hind paw of athymic BALB/c nu/nu mice. Subcutaneous injection of 3 mg/kg A-317491 or 3 mg/kg AF-353 was performed daily from day 10 through day 28 post-inoculation. Mouse hind paw volume was measured by using a plethysmometer (IITC Life Science) (Additional file 1: Figure S1C).

#### Tongue SCC model

The tongue SCC model was produced in athymic BALB/c nu/nu mice as previously described [22]. The anatomic and functional features of this mouse cancer model parallel those found in HNSCC patients [22]. After baseline Dolognawmeter gnaw times were established, BALB/c nude mice were inoculated with 50 μl total volume (of 10^6^) HSC-3 cells in DMEM and Matrigel™ into the tongue through a transoral approach. Control mice received 50 μl of vehicle injection. 20 μl of an ATP-hydrolyzing enzyme apyrase (100 μM, Sigma-Aldrich), A-317491 (3 mg/kg, Sigma-Aldrich), AF-353 (3 mg/kg, Afferent Pharmaceuticals) or normal saline solution was injected into the tongue of mice on post-inoculation day 14.

### Behavioral assessment

#### Paw withdrawal assay

Testing was performed by an observer blinded to the experimental groups. The paw withdrawal threshold was measured by an electronic *von Frey* anesthesiometer (IITC Life Sciences). Paw withdrawal threshold was defined as the force (g) sufficient to elicit a distinct paw withdrawal flinch upon application of the probe tip. A mean of eight withdrawal thresholds was calculated.

#### Orofacial function measurement

Behavioral testing with the Dolognawmeter was performed as previously described [29]. Briefly, each animal was placed in a tube in which access to escape was obstructed by a series of two polymer dowels. The animal voluntarily gnaws through the two dowels to escape from confinement within the tube. Each polymer dowel is connected to a digital timer. When a bar is severed by the mouse, a timer dedicated to the respective dowel is stopped. Animals were trained for 10 gnawing trials and then a baseline gnaw-time was established for each animal. Gnawing behavior was measured 15 minutes after drug treatment on post-inoculation 14. Data were analyzed as percent change of gnaw-time from baseline for each mouse.

### Immunofluorescence

Coverslips with plated neurons were fixed with 4% PFA and blocked with superblock (Thermo Fisher Scientific) for 30 min. Human HNSCC tissues were resected and fixed with 4% PFA, dehydrated, embedded in paraffin, and cut into 8-μm sections. Sections were then deparaffinized and blocked with superblock. H&E staining was performed to confirm cancer lesions. For immunofluorescence labeling, neurons or tissue sections were then incubated for 24 h at 4°C in rabbit anti-P2X3 (1:500, Alomone Labs) and goat anti-P2X2 (1:500, Santa Cruz Biotechnology). The sections were then washed in phosphate-buffered saline (PBS) with Triton X-100 and incubated in secondary antibody chicken anti-rabbit Alexa-594 (1:1000, Invitrogen) and donkey anti-goat Alexa-488 (1:1000, Invitrogen) in a dark chamber for 2 hours at room temperature. Control experiments were performed by incubation in secondary antibody alone and by applying P2X2 blocking peptides (Santa Cruz Biotechnology), and P2X3 blocking peptides (Alomone Labs). The coverslips or sections were washed and visualized with images acquired using a Nikon Ti Eclipse microscope (Nikon).

### Microdialysis

Mice were anesthetized with ketamine/xylazine (9:1, vol/vol). A microdialysis probe (CMA30, CMA-Microdialysis) was inserted through a guide cannula into the tongue of mice with tongue SCC or normal mice. Tongues were perfused with PBS at a constant flow rate of 1.0 μl/min using a CMA-402 microsyringe pump. After a 30 minute equilibration period, samples were collected for 90 minutes and kept at 4°C. Six mice were used in each group.

### ATP Luminescence assay

ATP concentration in the mouse tongue microdialysate samples and HNSCC supernatant were determined using ENLITEN ATP assay kit (Promega). Luminescence intensity was determined using a luminometer (GloMax-Multi Detection System, Promega). Calibration curves were obtained using standard ATP samples with subtraction of background luminescence of PBS. For SCC supernatant, 5×10^4^, 10^5^, 2×10^5^ cells were seeded onto separate culture plates and incubated in 3 ml of serum-free medium. Media was collected after 12 hours of incubation and ATP quantification was performed immediately.

### Calcium imaging

Cultured TG neurons were loaded with 1 μM of the cell permeable calcium sensitive dye, Fura 2 AM (Molecular Probes) for 30 min and washed with HBSS before use. Coverslips containing neurons were placed in a chamber with constant infusion of phenol-red free DMEM at room temperature. Fluorescence was detected by a Nikon Eclipse TI microscope (Nikon) fitted with a 20x fluor/NA 0.75 objective lens. Fluorescence images of 340 and 380 excitation wavelengths were collected and analyzed with the TI Element Software (Nikon). For drug treatments, neurons were pre-incubated with either AF-353 (1 μM), or A-317491 (1 μM) for 20 min prior to SCC supernatant application. Cells were counted as SCC supernatant responsive if the 340/380 ratio is ≥0.2 from baseline.

### Electrophysiology

Coverslips with neurons were transferred to a recording chamber and perfused continuously with external solution containing the following (in mM): 140 NaCl, 4 KCl, 2 MgCl_2_, 2 CaCl_2_, 10 glucose and 10 HEPES (pH 7.3 adjusted with NaOH, 320 mOsm/kg with sucrose), at room temperature. Patch pipettes were double-pulled (P-2000, Sutter, CA) from quartz glass capillaries (Q100-50-10, Sutter). They were adjusted to 2–8 MΩ when filled with a pipette solution (in mM): KCl 145, MgCl_2_ 3, CaCl_2_ 2.25, EGTA 1, HEPES 10 (pH 7.3 adjusted with KOH, 310 mOsm). After establishing the whole-cell configuration, the voltage was clamped at −60 mV using Axopatch 200B amplifier (Axon Instrument) and controlled by Clampex software (pClamp 10.2; Axon Instrument). DMEM, SCC supernatant, and drugs were applied using a fast-step SF-77B perfusion system (Warner Instrument) with three-barreled pipette placed near the cell. Current amplitudes were measured at the peak of the inward component.

### qRT-PCR

Human oral SCC and anatomically matched, contralateral normal oral epithelium from 10 oral cancer patients were surgically removed and immediately snap frozen in liquid nitrogen and stored at −80°C. Fresh TG neurons from mice with tongue SCC or normal mice were collected and stored at −80°C (n = 8 in each group). Tissues were homogenized and total RNA isolation of each sample was conducted with a Qiagen AllPrep DNA/RNA Micro Kit (Qiagen Inc.). Reverse transcription was carried out with a High Capacity cDNA Reverse Transcription Kit (Applied Biosystems Inc.) according to the manufacturer’s instructions. Quantitative real-time PCR was performed with the Taqman Gene Expression Assay Kit (Applied Biosystems Inc.). Primers were purchased from Life Technologies (Mouse P2X3: Mn00523699_m1; Mouse P2X2: Mn00462952_m1). The housekeeping gene β-actin was used as the internal control gene. Relative quantification analysis of gene expression data was calculated using the 2 − ΔΔCt method.

### Statistical analysis

SigmaPlot 11.0 for Windows was used to perform the statistical analysis. Student’s *t*-test, paired *t*-test, one-way Analysis of Variance (ANOVA) with a Tukey multiple comparisons post-hoc test, two-way ANOVA, and regression analysis were used where appropriate. Significance level was set at *P < 0.05, **P < 0.01, ***P < 0.001. Results were presented as mean ± SEM.

## Results

### The human cancer microenvironment is characterized by (i) a high ATP concentration, which correlates with pain intensity, and (ii) innervation by nerves expressing P2X2 and P2X3 subunits

**Figure 1:**
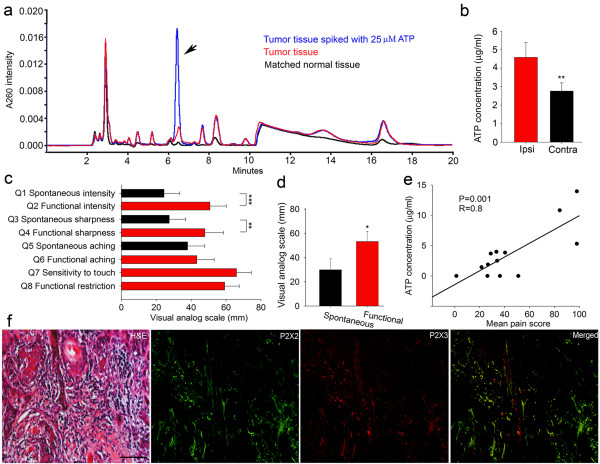
**ATP, P2X2, and P2X3 in human HNSCC microenvironment and pain. a**. Representative HPLC chromatograms showing ATP peaks from tumor and matched normal tissue harvested from the same patient. **b**. Tumor tissues (Ipsi) had higher levels of ATP compared to matched normal sites (Contra) (n = 10, Student’s *t*-test). **c**. Pain scores of functional sharpness and intensity were significantly higher than spontaneous sharpness and intensity, respectively (n = 13, Student’s *t*-test). **d**. Mean scores of functional pain (Q2, 4, 6, 7, 8) were significantly higher than spontaneous pain (Q1, 3, 5) (Student’s *t*-test). **e**. ATP concentration in extracted cancer tissue correlated positively with mean pain scores (linear regression). **f**. Representative H&E and immunofluorescence staining (P2X2, P2X3, merged) of a human tongue SCC. Sections were taken from adjacent sections of the SCC. Scale bar: 100 μm.

The ATP concentration in extracted HNSCC tissue was significantly higher than in anatomically matched healthy tissues from the same patients as measured by HPLC (Figure 1a and b). HNSCC patients reported both spontaneous and functional pain; however, functional pain was significantly higher than spontaneous pain (Figure 1c and d). Mean pain scores correlated positively with ATP concentration from extracted cancer tissue (Figure 1e). Patient demographic data, tumor location and staging are presented in Additional file 1: Table S1. We investigated whether P2X2 and P2X3 receptor subunits were expressed in the nerves innervating human HNSCC tissue. We observed intense P2X2 immunoreactivity on nerves associated with human HNSCC; the majority of these nerves also displayed immunoreactivity to our P2X3 probe (Figure 1f). HNSCC cells in patients’ tumor sections did not exhibit appreciable levels of P2X2 and P2X3 immunoreactivity (Additional file 2: Figure S1a).

### HNSCC secretes ATP which activates P2X2 and P2X3 subunits on TG neurons and produces pain

**Figure 2:**
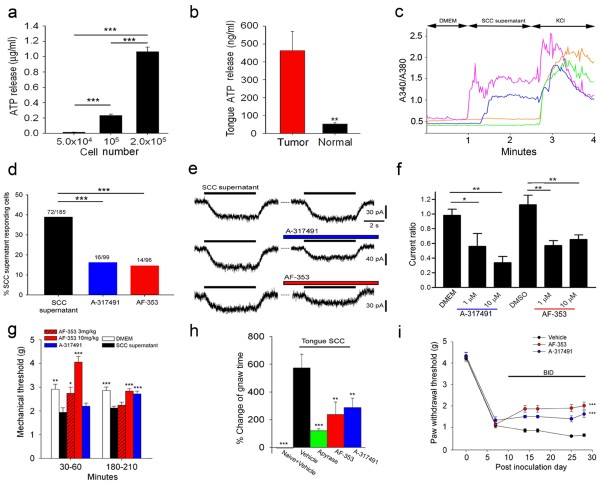
**ATP in the HNSCC microenvironment activates neurons and causes pain through P2X2/3 heterotrimers. a**. ATP concentration increases with HNSCC cell number (One-way ANOVA). **b**. ATP concentration in microdialysate of mouse tongue SCC was higher than normal mouse tongues (n = 6 in each group, Student’s *t*-test). **c**. Representative traces of calcium response (A340/A380) to culture media (DMEM), HNSCC supernatant, and 50 mM KCl (positive control). Each line represents one cell. **d**. Preincubation with A-317491 or AF-353 reduced the percentage of TG neurons responding to HNSCC supernatant based on calcium imaging (χ^2^-test). **e**. Representative whole cell recordings of TG neurons in response to HNSCC supernatant in presence and absence of A-317491 and AF-353. **f**. A-317491 and AF-353 reduced current ratio compared to vehicle controls in ATP-responding TG neurons (One-way ANOVA). **g**. HNSCC supernatant injection into the mouse hind paw caused mechanical nociception compared to DMEM injection. AF-353 reversed HNSCC supernatant-induced mechanical nociception measured at 30–60 minutes post injection. After 180–210 minutes, both antagonists reversed HNSCC supernatant-induced mechanical nociception (n = 7-9 in each group, One-way ANOVA compared to HNSCC supernatant group). **h**. Oral function was significantly impaired in tongue SCC mice compared to naïve mice. ATP hydrolyzing enzyme apyrase, AF-353 and A-317491 significantly improved oral function in HNSCC mice (n = 6-10 in each group; One-way ANOVA compared to HNSCC mice with vehicle treatment). **i**. In mice with paw SCC, twice daily (BID) treatment of AF-353 and A-317491 significantly reduced mechanical nociception compared to vehicle (n = 8 for each group; Two-way ANOVA).

HNSCC cells (HSC-3 cell line) secreted ATP spontaneously and the ATP concentration positively correlated with increasing cell number (Figure 2a). Microdialysate collected from mouse tongue HNSCCs *in situ* showed significantly elevated levels of extracellular ATP compared to microdialysate collected from normal tongues (Figure 2b). We next asked which receptors are activated by ATP released from HNSCC in mouse models. We characterized P2X2 and P2X3 on TG neurons based on their response to HNSCC supernatant using calcium imaging and electrophysiology. When we applied HNSCC supernatant to TG neurons, we observed an increased intracellular Ca^2+^ concentration in 39% (72/185) of TG neurons (Figure 2c-d). Application of two different classes of P2X3 and P2X2/3 receptor antagonists, a competitive antagonist A-317491 and an allosteric antagonist AF-353, resulted in a significant decrease in the percentage of neurons responding to HNSCC supernatant (Figure 2d): 16% (16/99) following A-317491 treatment, and 15% (14/96) following AF-353 treatment. We next used electrophysiology to characterize TG neurons following the application of ATP and HNSCC supernatant (Figure 2e-f). Whole-cell patch-clamp recording showed a sustained inward current in 52% (44/84) of cultured TG neurons (Figure 2e) and no transient current was recorded following HNSCC supernatant application. A-317491 dose-dependently inhibited the supernatant-induced current, while the inhibitory effect of AF-353 plateaued at 1 μM (Figure 2f).

To examine the effect of ATP, P2X2 and P2X3 receptors in HNSCC pain *in vivo*, we used three different mouse models of HNSCC. HNSCC supernatant injected into the mouse hind paw produced mechanical nociception (Figure 2g), which was fully reversed for 1 hour by intraplantar injection of 3 mg/kg AF-353, and for the full 3.5 hours with 10 mg/kg AF-353. Reversal of supernatant-induced pain with 3 mg/kg A-317491 was achieved for 3–3.5 hours after injection (Figure 2g). These results possibly reflected the differential rates of disposition following local injection of the polar A-317491 and lipophilic AF-353, respectively [14, 28, 30]. To examine the effect of ATP from cancer on orofacial sensory function, we inoculated HNSCC cells into the mouse tongue. Mice with tongue cancer exhibited significant orofacial nociception (*i.e.,* increased gnaw time) compared to control mice in a quantitative orofacial pain assay (Figure 2h) [29]. The ATP-hydrolyzing enzyme apyrase, AF-353 or A-317491 significantly reduced oral nociception in mice with HNSCC with a single intratumor injection (Figure 2h). To test the long-lasting effect of AF-353 and A-317491 on cancer growth and mechanical nociception, we inoculated HNSCC cells into the mouse hind paw and paw volume was quantified as an index for cancer growth. Systemically (subcutaneously) administered A-317491 and AF-353 over several weeks reduced HNSCC-induced mechanical nociception (Figure 2i), without affecting tumor growth (Additional file 2: Figure S1b).

### Co-culture in HNSCC induces P2X2/3 plasticity in TG neurons in a NGF-dependent manner

**Figure 3:**
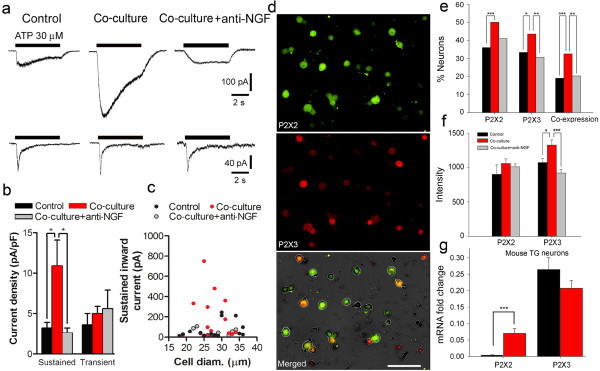
**HNSCC induces neuronal P2X2/3 plasticity that is reversed by anti-NGF. a**. Sustained ATP current (top panel) is enhanced and prolonged following SCC co-culture; anti-NGF added into the co-culture reduced the sustained ATP current. Transient ATP current (lower panel) is not affected by either co-culture or anti-NGF. **b**. Sustained ATP current density is increased by co-culture (One-way ANOVA), and is reversed by anti-NGF. **c**. Dot plot of sustained ATP current in different diameter neurons. Co-culture increased current in medium-sized TG neurons; this increased current was reversed by anti-NGF. **d**. Representative immunofluorescence images of P2X2 and P2X3 expression in TG neurons. **e**. HNSCC co-culture increased the percentage of neurons expressing P2X2 and P2X3 subunits. Anti-NGF treatment significantly reduced percentage of neurons expressing P2X3 but not P2X2 subunits. The significant increase in the percentage of neurons that express both subunits following co-culture was reversed by anti-NGF application (One-way ANOVA). **f**. P2X2 immunofluorescence intensity was not changed after co-culture or anti-NGF treatment. P2X3 immunofluorescence intensity was significantly increased following co-culture, and was reduced by anti-NGF (one-way ANOVA). **g**. In mice with tongue HNSCC, mRNA expression for P2X2 was increased, while P2X3 expression was unchanged in TG neurons (Student’s *t*-test).

To investigate the role of NGF on P2X receptors, we co-cultured HNSCC cells and mouse TG neurons. TG neurons exhibited both transient (P2X3 homotrimers) and sustained current (P2X2 homotrimers and/or P2X2/3 heterotrimers) in response to 30 μM ATP (Figure 3a). Following co-culture with HNSCC cells, sustained ATP current in some TG neurons was enhanced and prolonged, while properties of other TG neurons showing transient currents were unchanged (Figure 3a and 3b). Adding anti-NGF into the co-culture media significantly reduced amplitude and density of the sustained ATP current in TG neurons, without affecting the transient ATP current (Figure 3a and 3b). Measurement of the sustained current in TG neurons and reversal by anti-NGF as a function of cell diameter revealed that the responsive neurons were medium-sized (Figure 3c), *i.e*., those expressing both P2X2 and P2X3 subunits [7]. Other electrophysiological parameters of recorded neurons were not affected by co-culture or the addition of anti-NGF (Additional file 1: Table S2).

To further characterize specific P2X receptor subtypes in cancer pain, we stained cultured TG neurons using immunofluorescence (Figure 3d) and compared these results with patch-clamp recordings. In normal TG neuron culture, 14% of neurons exclusively expressed the P2X3 subunit, 17% of neurons exclusively expressed P2X2, and 19% neurons expressed both subunits (Figure 3e, Table 1). These observations corroborated the patch-clamp recordings, in which ATP application activated transient current in 17% neurons and sustained current in 34% neurons (Table 1). Co-culture significantly increased the percentage of neurons expressing both P2X subunits, from 19% at baseline to 33% after co-culture (Figure 3e, Table 1). Anti-NGF significantly reduced the percentage of co-cultured neurons expressing both P2X subunits (Figure 3e). Patch-clamp experiments showed that the number of cells exhibiting sustained ATP-induced current increased from 34% in normal neurons to 44% following co-culture (Table 1). Percentage of neurons exhibiting a transient ATP-induced current was similar between normal (17%) and co-cultured neurons (14%). Our electrophysiology results demonstrated that co-culture or anti-NGF only affected medium-sized neurons which contain both P2X2 and P2X3 subunits. Similarly, immunofluorescence revealed that co-culture increased, while anti-NGF reduced, the percentage of cells expressing P2X2 and P2X3 subunits on TG neurons (Figure 3e). Co-culture or anti-NGF treatment significantly affected P2X3 but not P2X2 immunofluorescence intensity (Figure 3f). P2X2 mRNA was significantly increased in TG neurons harvested from mice with tongue cancer compared to normal mice (Figure 3g), suggesting a chronic effect of HNSCC on P2X2 mRNA expression. No significant difference was observed for P2X3 mRNA expression between cancer and normal mice.

**Table 1:** **Comparison of percentage of cells in different culture conditions revealed by immunofluorescent labeling and patch clamping experiments**

TG Neuron culture condition	Immunofluorescence				Patch clamp recording	
	P2X3 only	P2X2 + P2X2/3			Transient current	Sustained current
		P2X2 only	P2X2 + P2X3	total	P2X3 only	P2X2 + P2X2/3
Normal	14%	17%	19%	36%	17%	34%
Co-culture	11%	17%	33%	50%	14%	44%
Co-culture + Anti-NGF	11%	21%	20%	41%	16%	49%

## Discussion

In the present report we elucidated a mechanism within the cancer microenvironment that drives cancer pain. We identified ATP as a potent algogenic mediator in both HNSCC patients and animal models. We evaluated differential roles of P2X2 and P2X3 subunits in HNSCC pain using calcium imaging, patch clamping, and molecular approaches. Using three different animal models of HNSCC pain with application of an ATP degrading enzyme, and two antagonists specific to P2X3 and P2X2/3 receptors, we confirmed the role of these receptors in HNSCC pain. Lastly, we demonstrated how other algogenic mediators like NGF, can modulate the plasticity of P2X2 and P2X3 subunits to amplify painful signals.

Evidence suggesting the involvement of ATP, P2X2 and P2X3 in pain signaling has been obtained mostly from rodent sensory systems at the level of the spinal cord and DRGs. Few studies have been conducted in trigeminal system. Furthermore, there has always been a question whether these findings in rodents can be translated into human physiology. A recent study by Serrano et al. reported that monkey and human DRG neurons do not express appreciable levels of P2X2 subunit, contrary to rodent sensory neurons [16]. The authors also demonstrated that monkey DRG neurons have functional P2X3 activity but lack functional P2X2/3 receptors. In transfected HEK293 cells, the authors found that the pharmacology of P2X3 receptors was different between rodents and primates. In our study, we report for the first time that the human HNSCC microenvironment contains high levels of ATP which correlates with patients’ pain intensity. We showed that in the human trigeminal system, both P2X3 and P2X2 subunits are expressed in the peripheral terminals of primary afferent neurons. Future studies need to confirm whether P2X2 and P2X3 subunits are expressed in normal tongue tissues, and whether pathological conditions like cancer lead to upregulation of P2X receptors in humans [15, 25]. Functional studies are also needed to compare trigeminal P2X2, P2X3, and P2X2/3 receptors between human and rodents.

Importantly, we identified P2X2/3 expressing neurons as a player in cancer pain, while neurons exclusively expressing P2X2 and P2X3 have minimal contributions. We showed that the human HNSCC microenvironment is innervated by nerve fibers exhibiting immunoreactivity to both of our P2X2 and P2X3 probes. Colocalization of P2X2 and P2X3 subunits in nerve fibers innervating the tongue strongly implicate the presence of P2X2/3 heterotrimeric receptors. We found that the ATP containing HNSCC supernatant induced a sustained current in mice trigeminal neurons, suggesting the involvement of P2X2 homotrimers and/or P2X2/3 heterotrimers. Co-culture of cancer cells with trigeminal neurons enhanced sustained ATP current in medium sized neurons, with no effect on transient ATP current (i.e. P2X3 receptors). Co-culture significantly increased the percentage of neurons expressing both P2X subunits. Consistent with previous studies [5], we found that these neurons co-expressing P2X2 and P2X3 subunits are medium sized. The effect of anti-NGF was also only observed in medium sized neurons exhibiting sustained current to ATP application, and co-expressing both P2X subunits. These collective results point out the importance of P2X2/3 heterotrimers in HNSCC pain and resonate with previous proposals that P2X2/3 receptors are major players in inflammatory and neuropathic models of chronic pain [10, 11, 31].

HNSCC pain is unique in its localization at the primary site; most other cancers hurt once they metastasize, usually to bone [2, 4]. Our data supports the peripheral mechanism of ATP and its receptors in HNSCC-induced mechanical nociception. First, high ATP concentrations in the peripheral human cancer tissues correlates with patients’ reported pain levels. Second, reducing ATP levels in HNSCC supernatant by the ATP degrading enzyme apyrase decreases HNSCC-induced mechanical nociception. Third, peripherally injected P2X2/3 antagonists also reduce HNSCC-induced mechanical nociception. Since A-317491 does not penetrate the blood–brain barrier [14], its anti-nociceptive effect is restricted to the periphery. It should be noted that although both AF-353 and A-317491 completely blocked HNSCC supernatant induced pain in the paw withdrawal assays, our calcium imaging, patch clamping, and two other orthotropic HNSCC models only demonstrated a partial role for P2X2/3 receptors. As both AF-353 and A-317491 predominantly antagonize P2X3 subunits [14, 28, 30], the observed remaining response could be gated through P2X2 subunits. Regardless of the receptors, our data strongly suggest that ATP serves as an important mediator in HNSCC-induced pain.

Our study demonstrated that cancer cells induce P2X2/3 plasticity with enhanced ATP sensitivity in trigeminal ganglia. Such plasticity is modulated by NGF, another major cancer-released mediator [22]. Two known modulators of P2X receptor sensitivity and expression are glial-derived nerve growth factor (GDNF) and NGF [18, 23, 24]. The proalgesic action of NGF is believed to be predominantly mediated by TrkA receptors with subsequent PKC activation and calcium release [32]. In trigeminal ganglia, TrkA colocalizes with P2X3 [5, 24]. We have previously shown that HNSCC produces NGF [22] but not GDNF [33], and further that NGF plays an important role in HNSCC proliferation and pain [22]. Using a pharmacologic approach (*i.e.,* anti-NGF treatment), we showed that NGF alters the expression levels of P2X2 and P2X3 subunits as demonstrated by whole-cell recordings and immunofluorescent staining. Increased receptor activity could also be explained by changes in membrane subunit composition or phosphorylation of the subunits, as proposed in other studies [24].

## Conclusions

We identified HNSCC as a major source of ATP in the HNSCC microenvironment and established the importance of heterotrimeric P2X2/3 receptors on trigeminal sensory fibers in HNSCC cancer pain. Our data also highlighted the importance of cancer cells in driving function-evoked pain and neuronal plasticity (*i.e.,* receptor upregulation and functional modulation) by tumorigenic mediators including NGF. We validated the ATP-P2X2/3 mechanism using preclinical animal models and human patients. Novel therapeutics that can potently antagonize P2X2/3 might have strong analgesic efficacy for human cancer pain.

## Electronic supplementary material

Additional file 1: **Tables and figure legend.** (DOCX 19 KB)

Additional file 2: **Figure.** (JPEG 2 MB)
