# Supplementary material for: Adenosine triphosphate drives head and neck cancer pain through P2X2/3 heterotrimers
Source: Acta Neuropathol Commun. 2014 Jun 5;2:62. doi: 10.1186/2051-5960-2-62 (PMC4229781; doi:10.1186/2051-5960-2-62)
Supplement: Supplementary file 1 — Additional file 1: Tables and figure legend. (DOCX 19 KB) [file 40478_2014_136_MOESM1_ESM.docx]

**Tables and Figure Legend**

**Supplemental Table 1. Patients’ demographic data, tumor location and staging**

| Mean (SE) age in years | Gender | Race | Tumor location | Staging |
| --- | --- | --- | --- | --- |
| 56.0 ± 10.3 | 6 M  7 F | 10 Caucasian  3 Asian | 8 tongue  2 gingiva  2 buccal mucosa  1 retromolar trigone | 3 T2N0M0  2 T2N2M0  3 T4N0M0  2 T4N0Mx  2 T2N2Mx  1 T2N0Mx |

**Supplemental Table 2. Electrophysiological properties of mice TG neurons (mean ± SE).**

|  | Control | Co-culture | Co-culture + anti-NGF |
| --- | --- | --- | --- |
| n | 41 | 36 | 37 |
| Diameter (µm) | 23.9 ± 0.8 | 24.4 ± 0.9 | 24.4 ± 0.9 |
| C_m_ (pF) | 17.8 ± 1.3 | 16.5 ± 1.2 | 18.8 ± 1.8 |
| R_in_ (MΩ) | 569 ± 109 | 531 ± 114 | 495 ± 124 |

**Supplemental Figure 1.** P2X2 and P2X3 receptors were not expressed in SCC and were not involved in SCC proliferation. a. H&E staining of human SCC (left panel). Immunofluorescent staining of adjacent sections showed that human SCC (in dotted line) do not express P2X2 (middle panel) or P2X3 (right panel) receptors. b. Sustained treatment with P2X2/3 antagonists (AF-353 and A-317491) in cancer mouse models did not affect SCC tumor size.
