# Supplementary figures and images for: Adenosine triphosphate drives head and neck cancer pain through P2X2/3 heterotrimers
Source: Acta Neuropathol Commun. 2014 Jun 5;2:62. doi: 10.1186/2051-5960-2-62 (PMC4229781; doi:10.1186/2051-5960-2-62)

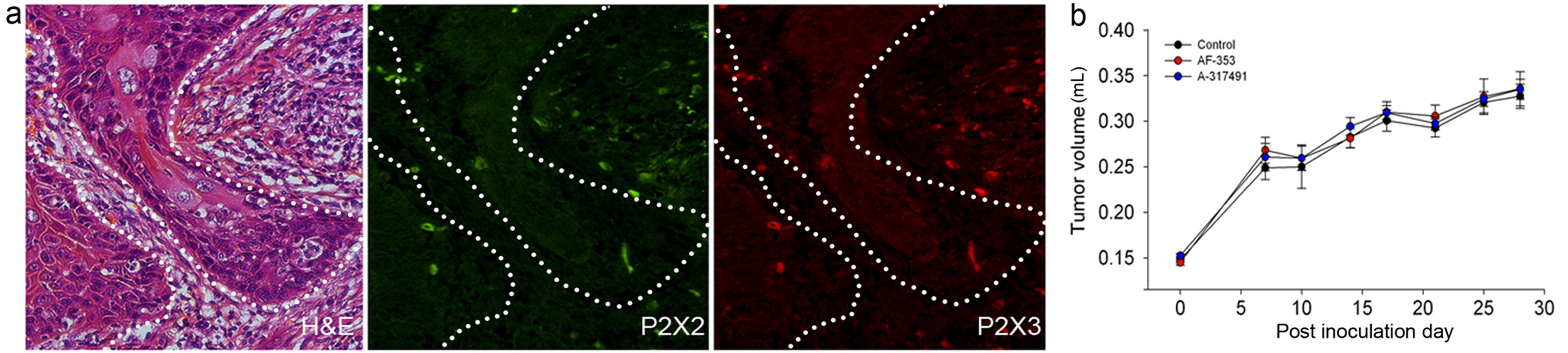

Supplement: Supplementary file 2 — Additional file 2: Figure. (JPEG 2 MB) [file 40478_2014_136_MOESM2_ESM.jpeg]
